# Biliary drainage prior to pancreatoduodenectomy with endoscopic ultrasound-guided choledochoduodenostomy versus conventional ERCP: propensity score-matched study and surgeon survey

**DOI:** 10.1055/a-2543-5672

**Published:** 2025-05-14

**Authors:** Jeska A. Fritzsche, Mike J. P. de Jong, Bert A. Bonsing, Olivier R. Busch, Freek Daams, Wouter J. M. Derksen, Lydi M. J. W. van Driel, Sebastiaan Festen, Erwin-Jan M. van Geenen, Frederik J. H. Hoogwater, Akin Inderson, Sjoerd D. Kuiken, Mike S. L. Liem, Daan J. Lips, Maarten W. Nijkamp, Hjalmar C. van Santvoort, Peter D. Siersema, Martijn W. J. Stommel, Niels G. Venneman, Robert C. Verdonk, Frank P. Vleggaar, Roeland F. de Wilde, Marc G. Besselink, Roy L. J van Wanrooij, Rogier P. Voermans, on behalf of the Dutch Pancreatic Cancer Group (DPCG), Dutch Pancreatic Cancer Audit (DPCA) and the pancreatoduodenectomy after EUS-CDS investigators, Foke van Delft, Joris I. Erdmann, Paul Fockens, Bas Groot Koerkamp, Geert Kazemier, Mark Meerdink, Wouter W. te Riele

**Affiliations:** 1 Department of Gastroenterology and Hepatology, Amsterdam UMC, location University of Amsterdam, Amsterdam, the Netherlands; 2 Amsterdam Gastroenterology Endocrinology Metabolism, the Netherlands; 3 Cancer Center Amsterdam, the Netherlands; 4 Department of Gastroenterology and Hepatology, Radboud University Medical Center, Nijmegen, the Netherlands; 5 Department of Surgery, Leiden University Medical Center, Leiden, the Netherlands; 6 Department of Surgery, Amsterdam UMC, location University of Amsterdam, Amsterdam, the Netherlands; 7 Department of Surgery, St Antonius Hospital, Nieuwegein, the Netherlands; 8 Department of Surgery, University Medical Center Utrecht, Utrecht, the Netherlands; 9 Department of Gastroenterology and Hepatology, Erasmus MC University Medical Center, Rotterdam, the Netherlands; 10 Department of Surgery, OLVG, Amsterdam, the Netherlands; 11 Department of Surgery, Universitair Medisch Centrum Groningen, Groningen, the Netherlands; 12 Department of Gastroenterology and Hepatology, Leiden University Medical Centre, Leiden, the Netherlands; 13 Department of Gastroenterology and Hepatology, OLVG, Amsterdam, the Netherlands; 14 Medisch Spectrum Twente, Department of Surgery, Enschede, the Netherlands; 15 Radboud University Medical Centre, Department of Surgery, Nijmegen, the Netherlands; 16 Medisch Spectrum Twente, Department of Gastroenterology and Hepatology, Enschede, the Netherlands; 17 St Antonius Hospital, Department of Gastroenterology and Hepatology, Nieuwegein, the Netherlands; 18 University Medical Centre Utrecht, Department of Gastroenterology and Hepatology, Utrecht, the Netherlands; 19 Department of Surgery, Erasmus MC Cancer Institute, University Medical Center, Rotterdam, the Netherlands; 20 Department of Gastroenterology and Hepatology, Amsterdam UMC, location Vrije Universiteit, Amsterdam, the Netherlands; 21 Department of Surgery,Amsterdam UMC, location Vrije Universiteit, Amsterdam, the Netherlands

## Abstract

**Background** Preoperative endoscopic biliary drainage may lead to complications (16 %–24 %), potentially hampering surgical exploration. Endoscopic ultrasound-guided choledochoduodenostomy (EUS-CDS) may reduce drainage-related complications; however, in the absence of surgeon-reported outcomes, it is unknown whether EUS-CDS may hamper surgical exploration. This study assessed the impact of preoperative EUS-CDS on pancreatoduodenectomy.

**Method** Consecutive patients who underwent pancreatoduodenectomy after preoperative biliary drainage were included in all eight centers performing EUS-CDS in the mandatory Dutch Pancreatic Cancer Audit (Jan 2020–Dec 2022). The primary outcome was major postoperative complications. Secondary outcomes included bile leak grade B/C, postoperative pancreatic fistula (POPF) grade B/C, and overall postoperative complications. A propensity score-matching (1:3) analysis was performed. Surgeons performing pancreatoduodenectomy after EUS-CDS completed a survey on surgical difficulty.

**Results** 937 patients with pancreatoduodenectomy after preoperative biliary drainage were included (42 EUS-CDS, 895 endoscopic retrograde cholangiopancreatography [ERCP]). Major postoperative complications occurred in 8 patients (19.0 %) in the EUS-CDS group and 292 (32.6 %) in the ERCP group (relative risk [RR] 0.50; 95 %CI 0.23–1.07). No significant differences were observed in overall complications (RR 0.95; 95 %CI 0.51–1.76), bile leak (RR 1.25; 95 %CI 0.31–4.98), or POPF (RR 0.62; 95 %CI 0.25–1.56). Results were similar after matching. The survey was completed for 29 pancreatoduodenectomies; surgery was not (13, 45 %), “slightly” (9, 31 %), “clearly” (5,17 %), and “severely” (2, 7 %) more complex because of EUS-CDS.

**Conclusion** This early experience suggests that preoperative biliary drainage with EUS-CDS does not increase the rate of complications after pancreatoduodenectomy and only infrequently hampers surgical exploration.

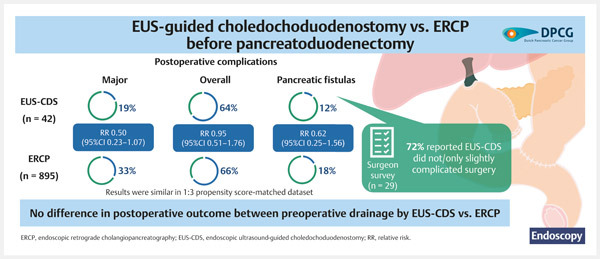

## Introduction

Patients with malignant distal biliary obstruction frequently require biliary drainage before undergoing pancreatoduodenectomy 1 . Traditionally, this is performed via endoscopic retrograde cholangiopancreatography (ERCP) with placement of a self-expanding metal stent (SEMS). This procedure is associated with a substantial risk of complications (range 16 %–24 %), especially post-ERCP pancreatitis (9 %–18 %) and reinterventions for stent-related problems (4 %–14 %), both potentially delaying and frustrating surgical exploration 2 3 4 5 6 7 . Moreover, these complications, especially post-ERCP pancreatitis, are associated with postoperative adverse events, prolonged hospital stay 8 9 , and delay or even cancellation of surgical treatment 4 8 9 10 .

In recent years, endoscopic ultrasound-guided choledochoduodenostomy (EUS-CDS) with a lumen-apposing metal stent (LAMS) has emerged as an alternative to ERCP in malignant distal biliary obstruction 11 . This approach is mostly used when ERCP fails or as a primary drainage modality in a trial setting, but it has also been suggested as a promising first-line alternative to ERCP 12 13 . Although several studies, including a randomized trial, have shown that EUS-CDS resulted in promising results in terms of technical success and adverse events, experience in resectable patients is still limited 11 12 13 14 . Most EUS-CDS series have focused on patients with unresectable or metastatic disease. In patients with resectable disease, endoscopists and surgeons have been reluctant to use EUS-CDS as data on the impact of perforations in both the duodenal and common bile duct wall on the surgical procedure are lacking and data on the risk of postoperative complications are scarce 12 15 16 17 18 19 .

The aim of this study was to assess the intraoperative and postoperative outcomes in patients who underwent pancreatoduodenectomy after preoperative biliary drainage by EUS-CDS compared with conventional biliary drainage by ERCP.

## Methods

### Study design

This study was a retrospective analysis of prospectively collected data from the Dutch Pancreatic Cancer Audit (DPCA) 20 . The DPCA is a mandatory audit for all hospitals performing pancreatic surgery in the Netherlands. Data were collected from all Dutch hospitals in which EUS-CDS was performed in a preoperative setting – five tertiary academic hospitals and three teaching hospitals. DPCA data from all patients who underwent pancreatoduodenectomy after preoperative endoscopic biliary drainage between January 2020 and December 2022 were included. Additional data from patients undergoing EUS-CDS and subsequent resection in 2023 were collected to expand the cohort. Patients who underwent percutaneous biliary drainage and patients with missing data on age, sex, and hospital of treatment were excluded from further analyses. The study protocol was approved by the scientific committee of the Dutch Pancreatic Cancer Group 21 . Given the observational character of the study, the Medical Ethics Review Committee confirmed that the Dutch Medical Research Act does not apply. In patients where additional data were requested and/or a surgical survey was performed, written informed consent was obtained.

### Biliary drainage procedures

Biliary drainage was performed when a multidisciplinary team considered it to be indicated. In general, biliary drainage was performed in patients with cholangitis, severe symptoms such as pruritus (caused by hyperbilirubinemia), severe hyperbilirubinemia (bilirubin concentration ≥ 250 µmol/L [≥ 14.6 g/dL]), mild jaundice (> 40 µmol/L [≥ 2.4 g/dL]) before administration of chemotherapy, or if the waiting time for surgery exceeded 3 weeks and it was anticipated that the bilirubin concentration would exceed ≥ 250 µmol/L (≥ 14.6 g/dL) at time of surgery. In the ERCP group, a fully covered SEMS was preferentially placed; the length of the stent was based on stricture characteristics and preference of the gastroenterologist. EUS-CDS was performed after an unsuccessful ERCP or as a primary drainage method in a research setting (SCORPION-p 14 and SCORPION-II-p 22 ), or in cases where biliary cannulation was considered impossible. For EUS-CDS, a Hot-Axios (Boston Scientific, Marlborough, Massachusetts, USA) 6 × 8 or 8 × 8 mm LAMS was used in seven centers, and a Niti-S (Hot-)NAGI (Taewoong Medical; Gimpo, South Korea) 10 × 20 mm LAMS was used in one center. A coaxial double-pigtail plastic stent or fully covered SEMS through the LAMS was used to prevent stent dysfunction, at the discretion of the endoscopist.

### Study outcomes and definitions

The primary outcome was the incidence of major postoperative complications, defined as Clavien–Dindo score ≥ 3 23 . Secondary outcomes were overall complications, pancreatic surgery-specific complications grade B/C (i. e. hepaticojejunostomy biliary leak, postoperative pancreatic fistula [POPF], delayed gastric emptying, post-pancreatectomy hemorrhage, and chyle leakage), pneumonia, surgical site infection, reinterventions, in-hospital mortality, hospital stay, and readmissions. All complications during hospital admission or up to 30 days after resection (in cases of earlier discharge) were registered. Pancreatic surgery-specific complications were all defined by the International Study Group of Pancreatic Surgery or the International Study Group of Liver Surgery 24 25 26 27 .

### Survey

Surgeons who performed pancreatoduodenectomy after EUS-CDS were requested to complete a five-question survey about the resection. This questionnaire consisted of questions about intraoperative findings related to the LAMS and potential surgical difficulties. The survey was intended to be sent on the same day as the resection; however, due to the delayed inclusion of additional centers, in some centers the survey was completed retrospectively. To assess potential recall bias and the influence of the lack of blinding, a sensitivity analysis was performed in surveys that were completed within 2 weeks after the resection.

### Statistical analysis

Normally distributed continuous patient and surgery characteristics data were summarized as mean with SD and compared using an independent *t* test. Non-normally distributed data were presented as median with interquartile range (IQR) and compared using a Mann–Whitney *U* test. The data distribution was evaluated through visual inspection. Categorical data were presented as frequencies with percentages and analyzed using the chi-squared test or Fisher’s exact test, as appropriate. A *P* value below 0.05 was considered statistically significant. Primary and secondary outcomes were presented as relative risk (RR) with corresponding 95 %CI or as mean differences with 95 %CI derived by bootstrapping with 5000 samples independently of the distribution of the variable 28 .

To minimize the impact of treatment allocation bias, patients from the EUS-CDS group were matched to patients from the ERCP group. Optimal pair matching was performed in a 1:3 ratio to increase power, without replacement. Variables for matching were selected based on baseline discrepancies and expected factors of influence on outcome. Baseline variables of sex, age, body mass index, American Society of Anesthesiologists score, liver- and pancreas-related comorbidities, tumor origin, neoadjuvant therapy, and hospital volume of more than 100 resections per year were identified as variables for the propensity score model. Only patients with a metal stent in the ERCP group were matched. To be able to calculate propensity scores for all patients, missing data for these variables (range 0–20 %) were imputed by multiple imputation and are reported in **Table 1 s** 29 . Only non-imputed data are presented in the manuscript; the imputed data are provided in **Table 2 s** . Covariate balance between treatment and control groups was assessed using the standardized mean difference (SMD) of the propensity score (distance). SMD values below 0.1 were considered indicative of acceptable balance. The overall SMD for the propensity score distance after matching was 0.066, suggesting adequate balance between the groups. Detailed balance diagnostics, consisting of the SMDs for individual covariates before and after matching, are provided in **Table 3 s** . Patients who eventually underwent EUS-CDS were included in the EUS-CDS group, and the ERCP group included patients who underwent successful ERCP with stent placement. An exploratory analysis was performed comparing 1) patients who underwent primary drainage attempt with EUS-CDS (without previous biliary cannulation attempt by ERCP or direct EUS-CDS in a clinical trial setting), and 2) patients who underwent a primary drainage attempt with ERCP. Statistical analyses were conducted with R software, version 4.2.1 (R Foundation for Statistical Computing, Vienna, Austria).

## Results

### Patient selection

**Fig. 1:**
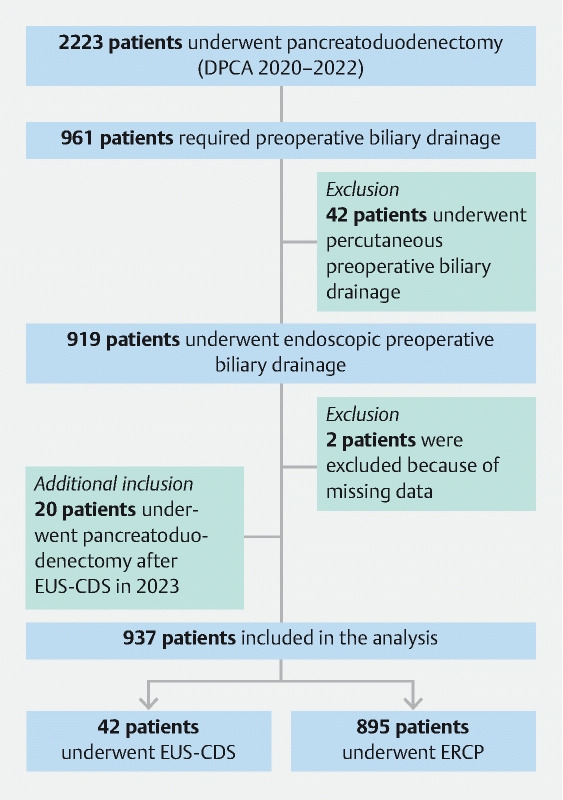
Flow chart of the screening and inclusion process. DPCA, Dutch Pancreatic Cancer Audit; EUS-CDS, endoscopic ultrasound-guided choledochoduodenostomy; ERCP, endoscopic retrograde cholangiopancreatography.

In total, 2243 patients underwent a pancreatoduodenectomy in the participating centers. Pancreatoduodenectomy following endoscopic biliary drainage was performed in 981 patients, of whom 937 were included in the analysis ( Fig. 1 **)** . EUS-CDS was performed as a rescue strategy after failed ERCP in most patients (n = 25, 59.5 %), while the other 17 patients (40.5 %) underwent primary drainage by EUS-CDS. Drainage was performed with LAMS alone in 20 patients (47.6 %), with placement of a coaxial double-pigtail plastic stent through the LAMS in 17 patients (40.5 %), and with placement of a fully covered SEMS through the LAMS in 4 patients (9.5 %). Coaxial stent placement was performed as a prophylactic measure (17, 81.0 %) or after stent dysfunction (4, 19.0 %). In one patient, EUS-CDS was performed using a fully covered SEMS after failed placement of the LAMS. In the ERCP group, SEMSs were placed in 633 patients (78.0 %) and plastic stents were placed in 179 patients (22.0 %); in 83 patients (9.3 %), information on the specific stent type was missing ( Table 1 ). Of the 633 SEMS placed, 512 were fully covered (80.9 %), 65 were uncovered (10.3 %), and this information was missing for 56 (8.8 %).

**Table 1:** Baseline characteristics of patients undergoing pancreatoduodenectomy after preoperative biliary drainage by endoscopic ultrasound-guided choledochoduodenostomy and endoscopic retrograde cholangiopancreatography.

	Unmatched cohort			Matched cohort		
	EUS-CDS (n = 42)	ERCP (n = 895)	*P*	EUS-CDS (n = 42)	ERCP (n = 126)	*P*
Age, mean (SD), years	67.7 (8.6)	67.1 (9.9)	0.65	67.7 (8.6)	68.5 (10.0)	0.63
Sex ratio M:F	21:21	497:398	0.59	21:21	64:62	> 0.99
BMI, mean (SD), kg/m ^2^	24.8 (5.5)	25.1 (4.1)	0.72	24.8 (5.5)	24.7 (3.7)	0.93
ASA score > 2	18 (42.9)	311 (35.1)	0.39	18 (42.9)	54 (43.5)	> 0.99
Comorbidity						
Liver cirrhosis	0 (0)	19 (2.1)	> 0.99 1	0 (0)	0 (0)	NA
Chronic pancreatitis	0 (0)	46 (5.1)	0.26 1	0 (0)	0 (0)	NA
Site of origin			**0.02** 1			0.98 1
Pancreas	28 (68.3)	504 (57.1)		28 (68.3)	88 (69.8)	
Distal bile duct	4 (9.8)	207 (23.5)		4 (9.8)	13 (10.3)	
Ampulla of Vater	5 (12.2)	146 (16.6)		5 (12.2)	14 (11.1)	
Duodenum or other	4 (9.8)	25 (2.8)		4 (9.8)	11 (8.7)	
Neoadjuvant therapy	10 (23.8)	240 (33.2)	0.28	10 (23.8)	27 (26.7)	0.88
Chemoradiotherapy	2 (4.8)	86 (11.9)		2 (4.8)	12 (11.9)	
Chemotherapy	8 (19.0)	150 (20.7)		8 (19.0)	14 (13.9)	
Radiotherapy	0 (0)	1 (0.1)		0 (0)	0 (0)	
Other	0 (0)	3 (0.4)		0 (0)	1 (1.0)	
Time to neoadjuvant therapy, 2 median (IQR), days	26 (24–31)	30 (20–42)	0.40	26 (24–31)	32 (20–45)	0.30
Type of stent	NA		NA	NA		NA
Metal		633 (78.0)			126 (100)	
Plastic		179 (22.0)			0 (0)	
Hospital volume > 100 per year 3	26 (61.9)	350 (39.1)	**0.005**	26 (61.9)	72 (57.1)	> 0.99

ASA, American Society of Anesthesiologists; BMI, body mass index; EUS-CDS, endoscopic ultrasound-guided choledochoduodenostomy; ERCP, endoscopic retrograde cholangiopancreatography; IQR, interquartile range; NA, not applicable.

Values are n (%) unless otherwise indicated. Percentages are calculated based on the total number of cases excluding missing values. The number of missing values for each characteristic is provided in **Table 1 s** . Bold values indicate statistical significance at the 5 % level.

1 Fisher’s exact test.

2 Only in patients in whom biliary drainage was performed prior to the start of neoadjuvant therapy: unmatched cohort EUS-CDS n = 9, ERCP n = 165; matched cohort ERCP n = 15.

3 Hospital volume was based on the mean total annual volume of pancreatoduodenectomy performed during the study period.

### Patient characteristics

No significant differences were present in baseline characteristics between the EUS-CDS group and ERCP group, except for the site of tumor origin ( *P* = 0.02) and hospital volume ( *P* = 0.005). After matching, overall balance was obtained in the baseline characteristics ( Table 1 ).

### Surgical characteristics

Surgical characteristics were comparable between the two groups, except for time between biliary drainage and surgery in patients without neoadjuvant therapy (median 32 days [IQR 22–39.25] in the EUS-CDS group vs. 41 days [IQR 28–54] in the ERCP group; *P* = 0.01) and operative time (median 309 minutes [IQR 245.75–353] in the EUS-CDS group vs. 349 minutes [IQR 281–425] in the ERCP group; *P* = 0.002). These differences remained after matching (43 days [IQR 28–54.75], *P* = 0.02; 363 minutes [IQR 293–437], *P* = 0.002). Intraoperative variables before and after matching are reported in Table 2 .

**Table 2:** Surgical characteristics of patients undergoing pancreatoduodenectomy after preoperative biliary drainage by endoscopic ultrasound-guided choledochoduodenostomy and endoscopic retrograde cholangiopancreatography.

	Unmatched cohort			Matched cohort		
	EUS-CDS (n = 42)	ERCP (n = 895)	*P*	EUS-CDS (n = 42)	ERCP (n = 126)	*P*
Time to surgery, 1 median (IQR), days	32 (22–39.25)	41 (28–54)	**0.01**	32 (22–39.25)	43 (28–54.75)	**0.02**
Type of resection			0.62 2			0.93 2
PRPD	30 (71.4)	583 (65.4)		30 (71.4)	86 (68.3)	
PPPD	11 (26.2)	289 (32.4)		11 (26.2)	37 (29.4)	
Other	1 (2.4)	20 (2.2)		1 (2.4)	3 (2.4)	
Minimally invasive surgery 3	6 (14.3)	199 (22.6)	0.28	6 (14.3)	22 (17.5)	0.81
Vascular resection 4	12 (28.6)	160 (18.9)	0.18	12 (28.6)	26 (21.1)	0.44
Arterial	1 (2.4)	31 (3.5)		1 (2.4)	6 (4.8)	
Venous	11 (26.2)	137 (16.1)		11 (26.2)	20 (16.0)	
Additional organ resection 5	5 (11.9)	86 (9.7)	0.59 2	5 (11.9)	16 (12.7)	> 0.99 ^2^
Dilated pancreatic duct (≥ 5 mm)	15 (38.5)	246 (32.4)	0.54	15 (38.5)	33 (31.4)	0.55
Pancreatic texture			0.24			0.56
Normal/soft	15 (41.7)	411 (53.0)		15 (41.7)	53 (49.1)	
Fibrotic/hard	21 (58.3)	364 (47.0)		21 (58.3)	55 (50.9)	
Blood loss, median (IQR), mL	310 (200–600)	500 (200–900)	0.14	310 (200–600)	500 (250–800)	0.13
Operative time, median (IQR), minutes	309 (245.75–353.50)	349 (281–425)	**0.002**	309 (245.75–353.50)	363 (293–437)	**0.002**
R0 resection 6	15 (39.5)	443 (55.5)	0.08	15 (39.5)	51 (43.2)	0.83

EUS-CDS, endoscopic ultrasound-guided choledochoduodenostomy; ERCP, endoscopic retrograde cholangiopancreatography; IQR, interquartile range; PRPD, pylorus-resecting pancreatoduodenectomy; PPPD, pylorus-preserving pancreatoduodenectomy.

Values are n (%) unless otherwise indicated. Percentages are calculated based on the total number of cases excluding missing values. The number of missing values for each characteristic is provided in **Table 1 s** . Bold values indicate statistical significance at a 5 % level.

1 Only in patients without neoadjuvant therapy. Unmatched cohort: EUS-CDS n = 32, ERCP n = 458; matched cohort: ERCP n = 70.

2 Fisher’s exact test.

3 Laparoscopic or robot, including patients with conversion to open surgery.

4 Vascular resection was reported according to the International Study Group for Pancreatic Surgery (ISGPS) classification 30 .

5 Including spleen (intentional or non-intentional), mesocolon transversum, colon segment, hemicolectomy, gastric resection, or other.

6 Resection margin status was classified as microscopically radical (> 1 mm; R0) or microscopically irradical (≤ 1 mm; R1) 31 .

In the 17 patients who underwent EUS-CDS as a primary drainage method compared with the 920 patients who underwent primary ERCP, median time to surgery was 22 days (IQR 20–36) vs. 41 days (IQR 28–54; *P* = 0.01) and operative time was 306.5 minutes (IQR 211–341.5) vs. 347.5 minutes (IQR 281–425; *P* = 0.006) ( **Table 4 s** ).

### Complications

Major postoperative complications occurred in 8 patients (19.0 %) in the EUS-CDS group and 292 patients (32.6 %) in the ERCP group (RR 0.50; 95 %CI 0.23 to 1.07). When including all complications after surgery, 27 patients (64.3 %) experienced at least one complication in the EUS-CDS group vs. 586 patients (65.5 %) in the ERCP group (RR 0.95; 95 %CI 0.51 to 1.76). POPF occurred in 5 patients (11.9 %) in the EUS-CDS group and 162 patients (18.1 %) in the ERCP group (RR 0.62; 95 %CI 0.25 to 1.56) ( Table 3 ). In the EUS-CDS group, fewer postoperative reinterventions (n = 6, 14.3 %) were performed compared with the ERCP group (n = 281, 31.4 %; RR 0.38; 95 %CI 0.16 to 0.89). This difference remained but was not significant in the matched cohort (RR 0.46; 95 %CI 0.21 to 1.08). Other secondary outcomes and type of reinterventions were comparable between the groups ( Table 3 ).

**Table 3:** Postoperative outcome of patients undergoing pancreatoduodenectomy after preoperative biliary drainage by endoscopic ultrasound-guided choledochoduodenostomy and endoscopic retrograde cholangiopancreatography.

	Unmatched cohort			Matched cohort		
	EUS-CDS (n = 42)	ERCP (n = 895)	RR or SMD (95 %CI)	EUS-CDS (n = 42)	ERCP (n = 126)	RR or SMD (95 %CI)
Major postoperative complication	8 (19.0)	292 (32.6)	0.50 (0.23 to 1.07)	8 (19.0)	40 (31.7)	0.59 (0.29 to 1.18)
Any postoperative complication	27 (64.3)	586 (65.5)	0.95 (0.51 to 1.76)	27 (64.3)	85 (67.5)	0.90 (0.52 to 1.55)
Postoperative pancreatic fistula, grade B/C	5 (11.9)	162 (18.1)	0.62 (0.25 to 1.56)	5 (11.9)	24 (19.0)	0.77 (0.36 to 1.65)
Delayed gastric emptying, grade B/C	6 (14.3)	165 (18.4)	0.75 (0.32 to 1.74)	6 (14.3)	20 (15.9)	0.91 (0.43 to 1.94)
Post-pancreatectomy hemorrhage, grade B/C	1 (2.4)	65 (7.3)	0.32 (0.05 to 2.31)	1 (2.4)	7 (5.6)	0.49 (0.08 to 3.11)
Hepaticojejunostomy biliary leak, grade B/C	2 (4.8)	34 (3.8)	1.25 (0.31 to 4.98)	2 (4.8)	7 (5.5)	0.88 (0.25 to 3.09)
Chyle leak, grade B/C	4 (9.5)	55 (6.1)	1.57 (0.58 to 4.24)	4 (9.5)	11 (8.7)	1.07 (0.44 to 2.60)
Pneumonia	2 (4.8)	30 (3.4)	1.41 (0.36 to 5.60)	2 (4.8)	5 (4.0)	1.15 (0.35 to 3.83)
Surgical site infection	7 (16.7)	141 (15.8)	1.07 (0.48 to 2.35)	7 (16.7)	6 (4.8)	1.93 (0.95 to 3.91)
Intensive care unit admission	4 (9.5)	62 (6.9)	1.39 (0.51 to 3.77)	4 (9.5)	9 (7.1)	1.26 (0.53 to 2.97)
Reintervention	6 (14.3)	281 (31.4)	**0.38 (0.16 to 0.89)**	6 (14.3)	39 (31.0)	0.46 (0.21 to 1.08)
Endoscopic	1 (2.4)	60 (6.7)	0.35 (0.05 to 2.50)	1 (2.4)	8 (6.3)	0.43 (0.07 to 2.79)
Radiological	5 (11.9)	223 (24.9)	0.42 (0.17 to 1.06)	5 (11.9)	34 (27.0)	0.45 (0.19 to 1.06)
Reoperation	2 (4.8)	58 (6.5)	0.73 (0.18 to 2.95)	2 (4.8)	8 (6.3)	0.79 (0.22 to 2.81)
In-hospital mortality	1 (2.4)	20 (2.2)	1.06 (0.15 to 7.37)	1 (2.4)	4 (3.2)	0.80 (0.14 to 4.68)
Length of hospital stay, 1 days						
Mean (95 %CI)	13.3 (10.4 to 18.8)	14.8 (14.0 to 16.0)	–1.5 (–4.6 to 3.9)	13.3 (10.4 to 18.8)	15.6 (13.6 to 18.2)	–2.3 (–6.1 to 3.1)
Median (IQR)	8.5 (6–15.25)	10 (7–16)	0.127 ^2^	8.5 (6–15.25)	11 (7–17.25)	0.078 2
Readmission within 30 days after discharge	8 (19.0)	142 (15.9)	1.23 (0.58 to 2.61)	8 (19.0)	17 (13.5)	1.35 (0.71 to 2.56)

EUS-CDS, endoscopic ultrasound-guided choledochoduodenostomy; ERCP, endoscopic retrograde cholangiopancreatography; IQR, interquartile range; RR, relative risk; SMD, standardized mean difference.

Values are n (%) unless otherwise indicated. Bold values indicate statistical significance at a 5 % level.

1 Length of hospital stay: unmatched cohort EUS-CDS n = 40, ERCP n = 863; matched cohort ERCP n = 121.

2 *P* value derived by Mann-Whitney U test.

In the 17 patients who underwent EUS-CDS as the primary drainage method, major postoperative complications occurred in 1 patient (5.9 %) compared with 299 patients (32.5 %) in the primary ERCP group (RR 0.13; 95 %CI 0.02 to 1.00). Ten patients (58.8 %) experienced at least 1 postoperative complication after primary drainage with EUS-CDS vs. 603 (65.5 %) who underwent primary drainage with ERCP (RR 0.76; 95 %CI 0.29 to 1.97). None of the 17 patients who underwent primary EUS-CDS developed a grade B/C POPF. Median length of hospital stay was 8 days (IQR 5–9) in the EUS-CDS group vs. 10 days (IQR 7–16.75) in the primary ERCP group ( *P* = 0.01) ( **Table 5 s** ).

### Survey

**Fig. 2:**
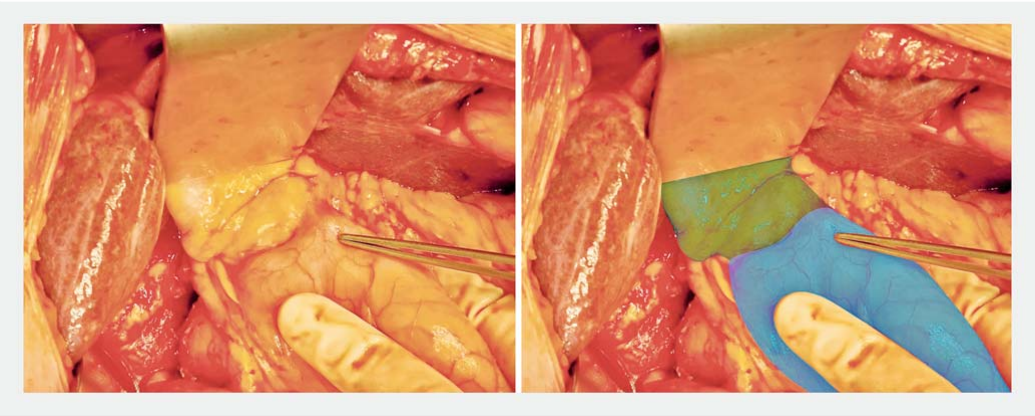
Intraoperative image of endoscopic ultrasound-guided choledochoduodenostomy. Green, bile duct; blue, duodenum.

The survey was sent to surgeons who recently performed 31 pancreatoduodenectomies after EUS-CDS. The survey was completed by 15 surgeons from eight hospitals regarding 29 procedures (response rate 94 %). The results of the survey are depicted in Table 4 . In the majority of procedures (n = 22, 76 %), surgeons did not visualize the stent but did notice the presence of the stent (n = 20, 69 %). In most procedures, surgeons noted some infiltration (n = 8, 28 %), fibrosis (n = 1, 3 %), or edema (n = 1, 3 %). In other patients, surgeons could palpate the stent (n = 5, 17 %), only noticed the stent when dissecting the bile duct (n = 2, 7 %), or noticed the adhesion of bile duct to the duodenum (n = 3, 10 %) ( Fig. 2 ). In most cases, surgery was not more complex (n = 13, 45 %) or only slightly more complex (n = 9, 31 %), due to inflammation (n = 4), adhesions (n = 3), or fibrosis (n = 2). In five patients (17 %), the surgery was clearly more complex, due to inflammation or infiltration (n = 3) or adhesions (n = 1), with one surgeon describing a more complex surgery without specifying the possible cause. In one of these five patients a major postoperative complication occurred. In two patients the surgeon described that the surgery was severely hampered. In both cases severe inflammation was present, which was presumed to be caused by the stent. In one of these resections, unintentional clamping injury of the proper hepatic artery was observed, and in the other, an aberrant artery was described that hindered the procedure in combination with the inflammation, leading to severe intraoperative bleeding. In all patients the distance between the LAMS and the hilum was sufficient to create a hepaticojejunostomy. The operative plan was altered in three patients due to the presence of the EUS-CDS. One of these patients is described above and in two others pancreatoduodenectomy was performed with pylorus ring resection rather than a pylorus-preserving procedure. Surveys were completed a median of 53 days (IQR 1–160 days) after the resection. Exploratory sensitivity analysis was conducted for surveys completed within 14 days after the resection (n = 14). In this group, surgeons reported a “not” (n = 5) or only “slightly” (n = 6) complicated surgery due to the presence of the stent ( **Table 6 s** ).

**Table 4:** Surgeon survey following pancreatoduodenectomy in patients with prior endoscopic ultrasound-guided choledochoduodenostomy.

Survey questions	EUS-CDS (n = 29)
Did you visualize the stent during the resection?	
Yes	7 (24)
No	22 (76)
Did you notice the presence of the stent during the resection?	
Yes	20 (69)
No	9 (31)
To what extent was the surgery complicated by the stent?	Median 2 (IQR 1–2)
1 – not complicated	13 (45)
2 – slightly complicated	9 (31)
3 – clearly complicated	5 (17)
4 – severely complicated	2 (7)
5 – impossible	0 (0)
Was there enough space between the hilum and the stent for the establishment of the hepaticojejunostomy?	
Yes	29 (100)
No	0 (0)
Did you have to adapt the surgical plan due to the presence of the stent?	
Yes	3 (10)
No	26 (90)

EUS-CDS, endoscopic ultrasound-guided choledochoduodenostomy; IQR, interquartile range.

Values are n (%) unless otherwise indicated.

When only patients with EUS-CDS as the primary drainage method were included (n = 12), two surgeons visualized the stent during a resection (17 %) and five (42 %) noticed infiltration, edema, or fibrosis, presumably caused by the stent. Two surgeons (17 %) only noticed the stent when dissecting the bile duct or adhesions to the duodenum. Surgery was not (n = 5, 42 %) or only slightly (n = 6, 50 %) hampered in the vast majority, and one surgeon (8 %) described clearly evidently hampered surgery due to inflammation.

## Discussion

This first propensity score-matched study including a surgeon survey on EUS-CDS found no increased risk of major complications after pancreatoduodenectomy in patients who underwent preoperative biliary drainage with EUS-CDS compared with conventional ERCP. In fact, patients undergoing EUS-CDS had a shorter time between biliary drainage and surgery and a shorter operative time. These results were similar after 1:3 propensity score matching. Only a few surgeons reported that EUS-CDS had a negative impact on surgical exploration.

Two previous studies compared the outcome of patients who underwent pancreatoduodenectomy after EUS-CDS vs. after ERCP. One retrospective French multicenter study assessed the impact of EUS-CDS in 44 patients on the rate of complications after pancreatoduodenectomy compared with ERCP in 112 patients from nine centers 19 . The authors reported that EUS-CDS was associated with fewer postoperative complications (77.3 % vs. 93.7 %) and shorter hospital stay (median 17 vs. 20 days) when compared with ERCP 19 . However, both SEMSs and plastic stents were included in the ERCP group, which may have blurred the study outcomes, as previous data have shown that postoperative outcome is better following SEMS placement than after plastic stent placement 7 . In order to neutralize this confounder, we performed a matched analysis in which only patients treated with SEMS were selected for the ERCP group. Moreover, our study is the first to include a surgeon survey, which adds interesting insight into potential difficulties caused by the stent that are not reflected in postoperative complications. We believe that the fact that our patients were matched, with only patients receiving a SEMS included in the matched analysis, and the addition of a surgeon survey substantially add to the currently available data and improve the implications of study outcomes.

In a randomized controlled trial (RCT), six patients undergoing pancreatoduodenectomy after EUS-CDS were compared with four patients who underwent preoperative ERCP 12 . A shorter operative time and hospital stay were reported after EUS-CDS, but this was not statistically significant given the very small sample size. Furthermore, two retrospective noncomparative studies have reported the postoperative outcomes of 5 and 21 patients, respectively, who underwent preoperative EUS-CDS 16 17 ; the latter study 17 is from the same group as the later French comparative study described above 19 ( **Table 7 s** ).

In contrast to this previous study 19 , we found no difference in (major) postoperative complications and hospital stay between the EUS-CDS and ERCP groups. No differences in individual adverse events could be identified in either of the studies 19 . When comparing our outcomes to studies on conventional endoscopic biliary drainage, the rate of postoperative major complications in the unmatched cohort (33 %) was somewhat higher when compared with a previous nationwide study from the Netherlands (24 %) and an RCT from Sweden comparing ERCP with SEMS and plastic stents (21 %) 3 7 . However, after excluding plastic stents in the matched cohort, results were comparable with the SEMS groups in both previous studies, showing the potential influence of including plastic stents on the study outcomes. More specifically, no difference was found in the rates of bile leak and POPF between the groups. A previous Dutch nationwide comparison between patients who underwent preoperative ERCP with either a plastic stent or SEMS reported less POPF with SEMS (9.8 % vs. 14.8 %) 7 . The higher risk of POPF is thought to be inversely correlated with fibrosis of the pancreas 32 33 . It was presumed that SEMS induces more pressure on the pancreatic duct, compared with plastic stents, leading to more pancreatic fibrosis and subsequently fewer POPF. Consequently, one might hypothesize that EUS-CDS could increase the risk of POPF compared with ERCP with LAMS, given that in EUS-CDS, the LAMS does not cause compression of the pancreatic duct. However, our data do not support this hypothesis, showing no difference in pancreatic texture nor risk of POPF, even after exclusion of patients who underwent plastic stent placement in the matched analysis. Moreover, it is worth mentioning that none of the patients who underwent EUS-CDS as primary drainage method developed POPF.

A potential benefit of EUS-CDS could be a shorter time between drainage and upfront surgery or time to neoadjuvant therapy, which was first reported by Janet et al. and further supported by our findings 19 . The shorter time to surgery after EUS-CDS may reflect less delay due to drainage-related complications. Preoperative complications caused by biliary drainage were not, however, part of the outcomes in the current study. This was done by intent as it has been clearly shown, even in RCTs, that preoperatively, EUS-CDS does not increase and possibly even reduces drainage-related complications, and the risk of reporting bias would have been significantly higher in this retrospective study 12 13 .

The observed similar postoperative outcomes do not, however, exclude the possibility that the surgical procedure becomes more technically challenging following EUS-CDS. Therefore, a surgeon survey was performed, which indeed showed that, in a subgroup of patients, EUS-CDS did complicate the procedure, presumably due to inflammation/infiltration or adhesions following the small intentionally made duodenal and biliary perforations. ERCP with SEMS placement may also cause some inflammation/infiltration or adhesions, even in the absence of clinically apparent pancreatitis. Currently, data on surgeon experience after ERCP with stent placement are unavailable, so we are unable to compare or even confirm this finding. It is nonetheless reassuring that most resections were not or only slightly hampered by the stent.

The results of this study should be interpreted considering several limitations. First, missing data were unavoidable due to the retrospective nature of the study. By using the prospectively collected data from the DPCA database, which is known for high quality data, we were able to limit the extent of missing data 20 . Second, only patients who underwent pancreatoduodenectomy were included, and therefore patients who had severe drainage-related complications who could not undergo surgery were not part of the study. Third, both patients who underwent primary EUS-CDS, as well as patients who first underwent an unsuccessful ERCP were included. This may have introduced bias as the attempted ERCP could have negatively influenced the results of the EUS-CDS group. Therefore, we performed an additional exploratory analysis including only patients who underwent EUS-CDS without a previous biliary cannulation attempt by ERCP. The analysis showed promising results with low (major) postoperative complication rates ( **Table 5 s** ), although no firm conclusion can be drawn due to the small number of patients and the lack of events in this group. Fourth, the fact that some of the surveys were completed retrospectively, potentially after the surgeon became aware of any complications, could have influenced the results. In an exploratory sensitivity analysis assessing surveys completed within 2 weeks, no surgeons reported a “clearly” complicated resection indicating the potential influence of the lack of blinding. Fifth, the sample size was relatively small, which means that the absence of differences in primary and secondary outcomes does not necessarily imply the absence of true differences. This study should therefore be considered an exploratory study, paving the way for larger prospective studies that also include patients with potentially resectable tumors. Sixth, we acknowledge that propensity score matching is vulnerable to residual confounding. In this study, propensity score matching was used as a sensitivity analysis, and no conclusions were drawn solely from the matched cohort. The consistency of outcome differences before and after matching suggests that baseline differences in observed variables were unlikely to drive these results. However, residual confounding cannot be excluded. The main strength of this study is its relatively large cohort, and the fact that it is the first study with propensity score matching comparing EUS-CDS with ERCP using only SEMS, and the first study with a surgeon survey to assess intraoperative findings due to EUS-CDS.

In conclusion, this study found that EUS-CDS was not associated with an increased rate of postoperative complications compared with SEMS placement by ERCP. Surgeons encountered no or minimal technical difficulties possibly related to EUS-CDS during the majority of resections. To confirm these findings and assess whether EUS-CDS may reduce the rate of post-biliary drainage complications, future randomized trials should specifically include patients with resectable tumors and report postoperative outcomes.

## Supplementary material

Supplementary material Zusatzmaterial
