# Supplementary material for: Biliary drainage prior to pancreatoduodenectomy with endoscopic ultrasound-guided choledochoduodenostomy versus conventional ERCP: propensity score-matched study and surgeon survey
Source: Endoscopy. 2025 May 14;57(7):719–29. doi: 10.1055/a-2543-5672 (PMC12204733; doi:10.1055/a-2543-5672)
Supplement: Supplementary file 1 — Supplementary material [file 24660supmat_10-1055-a-2543-5672.pdf]

Biliary drainage prior to pancreatoduodenectomy with endoscopic ultrasound-guided choledochoduodenostomy versus conventional ERCP: propensity score-matched study and surgeon survey

Jeska A. Fritzsche, Mike J. P. de Jong, Bert A. Bonsing, Olivier R. Busch, Freek Daams, Wouter J. M. Derksen, Lydi M. J. W. van Driel, Sebastiaan Festen, Erwin M. van Geenen, Frederik J. H. Hoogwater, Akin Inderson, Sjoerd D. Kuiken, Mike S. L. Liem, Daan J. Lips, Maarten W. Nijkamp, Hjalmar C. van Santvoort, Peter D. Siersema, Martijn W. J. Stommel, Niels G. Venneman, Robert C. Verdonk, Frank P. Vleggaar, Roeland F. de Wilde, Marc G. Besselink, Roy L. J van Wanrooij,*, Rogier P. Voermans,* on behalf of the Dutch Pancreatic Cancer Group and Dutch Pancreatic Cancer Audit†

*These authors share senior authorship.

†Working Group

Foke van Delft, Joris I. Erdmann, Paul Fockens, Bas Groot Koerkamp, Geert Kazemier, Mark Meerdink, Wouter W. te Riele

Table 1s Missing data in baseline and surgical characteristics of patients undergoing pancreatoduodenectomy after preoperative biliary drainage by EUS-CDS and ERCP.

	Unmatched cohort		Matched cohort	
	EUS-CDS (n=42)	ERCP (n=895)	EUS-CDS (n=42)	ERCP (n=126)
BMI	0 (0)	5 (0.6)	0 (0)	1 (0.8)
ASA score	0 (0)	8 (0.9)	0 (0)	2 (1.6)
Site of origin	1 (2.4)	13 (1.5)	1 (2.4)	0 (0)
Neoadjuvant therapy	0 (0)	172 (19.2)	0 (0)	25 (19.8)
Time to neoadjuvant therapy[a]	1 (10)	45 (21.4)	1 (10)	12 (44)
Type of stent	NA	83 (9.3)	NA	0 (0)
Time to surgery[b]	0 (0)	25 (5.2)	0 (0)	4 (5.4)
Type of resection	0 (0)	3 (0.3)	0 (0)	1 (0.8)
Minimally invasive	0 (0)	13 (1.5)	0 (0)	0 (0)
Vascular resection	0 (0)	47 (5.3)	0 (0)	3 (2.4)
Additional organ resection	0 (0)	5 (0.6)	0 (0)	0 (0)
Diameter pancreatic duct	3 (7.1)	136 (15.2)	3 (7.1)	21 (16.7)
Pancreatic texture	6 (14.3)	120 (13.4)	6 (14.3)	18 (14.3)
Blood loss	4 (9.5)	43 (4.8)	4 (9.5)	9 (7.1)
Operative time	2 (4.8)	103 (11.5)	2 (4.8)	20 (15.9)
R0-resection	4 (9.5)	97 (10.8)	4 (9.5)	8 (6.3)

Values are n (%). [a]Only in patients in whom biliary drainage was performed prior to the start of neoadjuvant treatment: 10 in EUS-CDS group and 210 in ERCP group in unmatched cohort and 27 in the matched cohort. [b]Only in patients without neoadjuvant therapy: 32 patients in EUS-CDS group and 483 patients in ERCP group in unmatched cohort and 74 patients in matched cohort. Abbreviations: ASA, American Society of Anesthesiologists; BMI, body mass index; ERCP, endoscopic retrograde cholangiopancreatography; EUS-CDS, endoscopic ultrasound guided choledochoduodenostomy.

Table 2s Imputed baseline variables in matched cohort.

Variables	Matched cohort		
	**EUS-CDS (n=42)**	**ERCP (n=126)**	**p-value**
BMI (kg/m^2), mean (SD)	24.8 (5.5)	24.7 (3.7)	0.931
ASA score >2	18 (42.9)	55 (43.7)	1.000
Site of origin			0.944[a]
Pancreas	28 (66.7)	88 (69.8)	
Distal bile duct	5 (11.9)	13 (10.3)	
Ampulla of Vater	5 (11.9)	14 (11.1)	
Duodenum or other	4 (9.5)	11 (8.7)	
Neoadjuvant therapy	10 (23.8)	31 (24.6)	1.000

Values are n (%) unless otherwise indicated. Bold values indicate statistical significance at a 5% level. [a]Fisher exact test. Abbreviations: ASA, American Society of Anesthesiologists; BMI, body mass index; ERCP, endoscopic retrograde cholangiopancreatography; EUS-CDS, endoscopic ultrasound guided choledochoduodeostomy; NA, not applicable; SD, standard deviation.

Table 3s Standardized mean differences (SMDs) for individual covariates before and after matching.

	Unmatched	Matched
Distance	0.620	0.066
Age	0.042	0.092
Sex	0.120	0.016
BMI	0.025	0.014
ASA >2	0.129	0.160
Comorbidity		
Liver cirrhosis	0.150	0.000
Chronic pancreatitis	0.222	0.000
Site of origin		
Pancreas	0.040	0.067
Distal bile duct	0.286	0.049
Ampulla of Vater	0.003	0.025
Duodenum or other	0.255	0.027
Neoadjuvant therapy	0.376	0.019
Hospital volume >100 per year	0.436	0.098

Abbreviations: ASA, American Society of Anesthesiologists; BMI, body mass index.

Table 4s Baseline and surgical characteristics by primary drainage attempt

Variables	Primary EUS-CDS (n=17)	Primary ERCP (n=920)	p-value
Age (years), median (IQR)	65 (55-73)	68 (61-74)	0.409
Sex ratio M:F	9:8	509:411	1.000
BMI (kg/m^2), median (IQR)	24 (22-27)	24 (22-27)	0.682
Missing	-	-	
ASA score >2	5 (29.4)	324 (35.5)	0.799[h]
Missing	-	*8*	
Comorbidity			
Liver cirrhosis	0 (0)	19 (2.1)	1.000[h]
Chronic pancreatitis	0 (0)	46 (5.0)	1.000[h]
Site of origin			0.756[h]
Pancreas	12 (70.6)	520 (57.4)	
Distal bile duct	2 (11.8)	209 (23.1)	
Ampulla of Vater	3 (17.6)	148 (16.3)	
Duodenum or other	0 (0)	29 (3.2)	
Missing	-	*14*	
Neoadjuvant therapy	4 (23.5)	246 (32.9)	0.602[h]
Chemoradiotherapy	1 (5.9)	87 (9.5)	
Chemotherapy	3 (17.6)	155 (16.8)	
Radiotherapy	0 (0)	1 (0.2)	
Other	0 (0)	3 (0.3)	
Missing	*0*	*172*	
Time to neoadjuvant therapy (days)[a], median (IQR)	22.5 (18.75-25.25)	30 (20-42)	0.144
Missing	*0*	*76*	
Hospital volume >100 per year[b]	15 (88.2)	361 (39.2)	**<0.001**
Time to surgery (days)[c], median (IQR)	22 (20-36)	41 (28-54)	**0.014**
Missing	-	*25*	
Type of resection			0.400[h]
PRPD	11 (64.7)	602 (65.6)	
PPPD	5 (29.4)	295 (32.2)	
Other	1 (5.9)	20 (2.2)	
Missing	-	*3*	
Minimally invasive[d]	2 (11.8)	203 (22.4)	0.389[h]
Missing	-	*13*	
Vascular resection[e]	5 (29.4)	167 (19.1)	0.347[h]
Arterial resection	0 (0)	32 (3.5)	
Venous resection	5 (29.4)	143 (16.3)	
Missing	0	47	
Additional organ resection[f]	2 (11.8)	89 (9.7)	0.678[h]
Missing	-	*5*	
Dilated pancreatic duct	7 (43.8)	254 (32.5)	0.495
	1	*138*	
Pancreatic texture			
Normal/soft	5 (38.5)	421 (52.8)	0.404[h]
Fibrotic/hard	8 (61.5)	377 (47.2)	

Missing	*4*	*122*	
Blood loss (mL), median (IQR)	400 (290-450)	500 (200-900)	0.442
Missing	*2*	*45*	
Operative time (min), median (IQR)	306.5 (211-341.5)	347.5 (281-425)	**0.006**
Missing	*1*	*104*	
R0-resection[g]	7 (41.2)	451 (55.1)	0.372
Missing	-	*101*	

Values are n (%) unless otherwise indicated. Bold numbers indicate statistical significance at a 5% level. [a]Only in patients in whom drainage was performed prior to the start of neoadjuvant treatment: 4 in EUS-CDS group and 170 in ERCP group. [b]Hospital volume was based on the mean total annual volume of pancreatoduodenectomy performed during the study period. [c]Only in patients without neoadjuvant therapy: 13 patients in EUS-CDS group and 502 patients in ERCP group. [d]Laparoscopic or robot, including patients with conversion to open surgery. [e]Vascular resection was reported according to the International Study Group for Pancreatic Surgery (ISGPS) classification [1]. [f]Including spleen (intentional or non-intentional), mesocolon transversum, colon segment, hemicolectomy, gastric resection, or other. [g]Resection margin status was classified as microscopically radical (>1 mm; R0) or microscopically irradical (≤1 mm; R1) [2]. [h]Fisher exact test. Abbreviations: ASA, American Society Anesthesiologists; BMI, body mass index; ERCP, endoscopic retrograde cholangiopancreatography; EUS-CDS, endoscopic ultrasound guided choledochoduodeostomy; IQR, interquartile range (P25-P75); PPPD, pylorus-preservering pancreatoduodenectomy; PRPD, pylorus-resecting pancreatoduodenectomy.

Table 5s Postoperative outcome by primary drainage attempt.

	Primary EUS-CDS (n=17)	Primary ERCP (n=920)	Relative Risk (95% CI) or p-value[a]
Major postoperative complication	1 (5.9)	299 (32.5)	**0.13 (0.02-0.996)**
Any postoperative complication	10 (58.8)	603 (65.5)	0.76 (0.29-1.97)
Postoperative pancreatic fistula, grade B/C	0 (0)	167 (18.2)	0.054[b]
Delayed gastric emptying, grade B/C	2 (11.8)	169 (18.4)	0.60 (0.14-2.59)
Post pancreatectomy hemorrhage, grade B/C	0 (0)	66 (7.2)	0.625[b]
Bile leakage, grade B/C	0 (0)	36 (3.9)	1.000[b]
Chyle leak, grade B/C	2 (11.8)	57 (6.2)	1.98 (0.46-8.47)
Pneumonia	0 (0)	32 (3.5)	1.000[b]
Surgical site infection	1 (5.9)	72 (7.8)	0.74 (0.10-5.50)
Intensive care unit admission	0 (0)	66 (7.2)	0.625[b]
Re-intervention	1 (5.9)	286 (31.1)	0.14 (0.02-1.06)
Endoscopic	0 (0)	61 (6.6)	0.620[b]
Radiological	1 (5.9)	227 (24.7)	0.19 (0.03-1.46)
Reoperation	0 (0)	60 (6.5)	0.619[b]
In-hospital mortality	0 (0)	21 (2.3)	1.000[b]
Length of hospital stay[a], median (IQR)	8 (5-9)	10 (7-16.75)	**0.012**[c]
Readmission within 30 days after discharge	2 (11.8)	148 (16.2)	0.70 (0.16-3.03)

Values are n (%) unless otherwise indicated. Bold values denote statistical significance at a 5% level. [a]Missing in 34 patients in ERCP group. [b]In case of zero events in one of the groups, a p-value was derived by Fishers exact test. [c]P-value derived by Wilcoxon rank sum test with continuity correction. Abbreviations: ERCP, endoscopic retrograde cholangiopancreatography; EUS-CDS, endoscopic ultrasound guided choledochoduodeostomy; IQR, interquartile range (P25-P75).

Table 6s Surgeon survey following pancreatoduodenectomy in patients with prior EUS-CDS (sensitivity analysis in surveys completed within 14 days after the resection).

Survey questions	N=11 EUS-CDS
Did you visualize the stent during the resection?	
Yes	2 (18)
No	9 (82)
Did you notice the presence of the stent during the resection?	
Yes	5 (45)
No	6 (55)
To what extent was the surgery complicated by the stent?	Median 2 (IQR 1-2)
1 - not complicated	5 (45)
2 - slightly complicated	6 (55)
3 - clearly complicated	0 (0)
4 - severely complicated	0 (0)
5 - impossible	0 (0)
Was there enough space between the hilum and the stent for the establishment of the hepaticojejunostomy?	
Yes	11 (100)
No	0 (0)
Did you have to adapt the surgical plan due to the presence of the stent?	
Yes	0 (0)
No	11 (100)

Values are n (%) unless otherwise indicated. Abbreviations: EUS-CDS, endoscopic ultrasound guided choledochoduodenostomy.

Table 7s Overview of previous studies reporting postoperative outcome of pancreatoduodenectomy in patients with prior EUS-CDS.

	Study	Design, geographic area	Period	Number of patients		Outcomes (EUS-CDS vs ERCP)							
				EUS-CDS	ERCP	Serious AEs	Overall AEs	POPF	HBL	Mortality	Hospital stay	Operative time	Time to surgery
1	Fabbri et al. 2019 [3]	Retrospective, single-centre, Europe	NR	5	NA	NR	NR	2 (40%)[a]	0 (0%)[a]	1 (20%)	NR	NR	NR
2	Gaujoux et al. 2021 [4]	Retrospective, multicentre, Europe	2016-2019	21 (7 primary)	NA	3 (14%)	17 (81%)[a]	2 (10%)[b]	0 (0%)[a]	0 (0%)	20 days [9-50]	355 min [180-650]	45 days [31-214][c]
3	Janet et al. 2023 [5]	Retrospective, multicentre, Europe	2015-2022	44 (28 primary)	112 (plastic/ SEMS)	NR	34 (77%)	12 (27%) vs 34 (30%)., p=0.85[a]	2 (5%) vs 5 (5%), p=1.00[a]	4 (9%) vs 3 (3%), p=0.09	17 days [13-22.5] vs 20 days [16-28.2], p=0.01	NR	28 days [17-38] vs 43 days [27.2-63], p=0.03[c]
4	Chen et al. 2023 [6]	RCT, multicentre, Canada and Europe	2019-2022	6 (all primary)	4	NR	NR	NR	NR	NR	7.4 days (1.5)	7.29h (2.22)	NR

[a]Grade unknown, [b]Only grade B/C, [c]Only in patients without neoadjuvant therapy. Abbreviations: AE, adverse event; ERCP, endoscopic retrograde cholangiopancreatography; EUS-CDS, endoscopic ultrasound guided choledochoduodenostomy; LAMS, lumen-apposing metal stent; NA, not applicable; NR, not reported; RCT, randomised controlled trial; SEMS, self-expanding metal stent.

SUPPLEMENTARY REFERENCES

1. Bockhorn M, Uzunoglu FG, Adham M, Imrie C, Milicevic M, Sandberg AA, et al. Borderline resectable pancreatic cancer: A consensus statement by the International Study Group of Pancreatic Surgery (ISGPS). Surgery. 2014;155(6):977-88.
2. The Royal College of Pathologists (2013) Standards and datasets for reporting cancers. Dataset for the histopathological reporting of carcinomas of the pancreas, ampulla of vater and common bile duct. The Royal College of Pathologists, London.
3. Fabbri C, Fugazza A, Binda C, Zerbi A, Jovine E, Cennamo V, et al. Beyond palliation: using EUS-guided choledochoduodenostomy with a lumen-apposing metal stent as a bridge to surgery. a case series. J Gastrointestin Liver Dis. 2019;28(1):125-8.
4. Gaujoux S, Jacques J, Bourdariat R, Sulpice L, Lesurtel M, Truant S, et al. Pancreaticoduodenectomy following endoscopic ultrasound-guided choledochoduodenostomy with electrocautery-enhanced lumen-apposing stents an ACHBT - SFED study. HPB (Oxford). 2021;23(1):154-60.
5. Janet J, Albouys J, Napoleon B, Jacques J, Mathonnet M, Magne J, et al. Pancreatoduodenectomy Following Preoperative Biliary Drainage Using Endoscopic Ultrasound-Guided Choledochoduodenostomy Versus a Transpapillary Stent: A Multicenter Comparative Cohort Study of the ACHBT-FRENCH-SFED Intergroup. Ann Surg Oncol. 2023;30(8):5036-46.
6. Chen YI, Sahai A, Donatelli G, Lam E, Forbes N, Mosko J, et al. Endoscopic Ultrasound-Guided Biliary Drainage of First Intent With a Lumen-Apposing Metal Stent vs Endoscopic Retrograde Cholangiopancreatography in Malignant Distal Biliary Obstruction: A Multicenter Randomized Controlled Study (ELEMENT Trial). Gastroenterology. 2023;165(5):1249-61.
